# Design and dynamic emulation of hybrid solar-wind-wave energy converter (SWWEC) for efficient power generation

**DOI:** 10.1038/s41598-024-72827-9

**Published:** 2024-09-30

**Authors:** Aryan Manan Jariwala, Santanu Kumar Dash, Umesh Kumar Sahu, Saichol Chudjuarjeen

**Affiliations:** 1 School of Mechanical Engineering, Vellore Institute of Technology, Vellore, 632014 India; 2 TIFAC-CORE, Vellore Institute of Technology, Vellore, 632014 India; 3 Department of Mechatronics, Manipal Institute of Technology, Manipal Academy of Higher Education, Manipal, Karnataka 576104 India; 4 Department of Electrical and Telecommunication, Rajamangala University of Technology, Krungthep, Bangkok, Thailand

**Keywords:** Power generation, Renewable energy, Solar-wind-wave energy converter, Wave energy, Energy science and technology, Engineering

## Abstract

As research into wave energy converters progresses and new developers enter the field, there arises a growing requirement for a standardized modelling approach. This article presents a novel design and dynamic emulation for a hybrid solar-wind-wave energy converter (SWWEC) which is the combination of three very well-known renewable energies: solar, wind and wave energy. Photovoltaic (PV) panels and vertical axis wind turbine (VAWT) are installed on top of the floating WEC that harness the energies from the sun and wind respectively. The SWWEC is designed with a point absorber capture system. An electrical motor is used to dynamically emulate the performance of the SWWEC under real world conditions to drive the DC generator. The present paper shows the importance and necessity of the required control schemes for the proper control of generator side converters which is present in the offshore marine substation and the most required grid connected onshore converters. The better switching signal generation for the converter control and generated harmonics elimination techniques are also presented in the paper. Outcomes of the present study are discussed and verified.

## Introduction

Amidst the escalating threat of global warming and the urgent need to address its catastrophic repercussions, there is a pressing demand for sustainable energy sources. The persistent reliance on finite and environmentally harmful fossil fuels underscores the necessity of transitioning to renewable energy alternatives. Among these, wave energy stands out as a promising yet underexploited resource with substantial potential for contributing to the global energy transition. By harnessing the kinetic energy present in the oceanic waves, wave energy conversion technologies offer a reliable and abundant source of green energy, with minimal environmental impact compared to conventional energy sources. The predictability and high energy density of oceanic waves make wave energy an attractive option for meeting the increasing energy demands of coastal regions globally. Researchers posit that oceanic waves have the capacity to generate 2 Terawatts (TW) annually on a global scale. The theoretical global wave energy potential is estimated at 8 × ${10}^{6}$ Terawatt hours (TWh) per year, surpassing the total hydroelectric generation worldwide by approximately 100-fold. In contrast, harnessing an equivalent amount of energy through fossil fuels would result in the emission of 2 million tons of CO2^1^. Over the past few decades, there has been ongoing progress in the advancement of technologies associated with harnessing and converting wave energy. This evolution has led to the emergence of various types of WECs^2^. Researchers from diverse global regions have proposed and developed a range of prototypes, with a particular emphasis on buoy design such as a, heaving buoy WEC^3^, design of the oscillating buoy WEC^4^ and structural optimization on the oscillating-array-buoys^5^ as they are currently undergoing testing and verification stages. A list of full-scale installed WECs across the globe are summarized in. "Laboratory Validation and Result Analysis" discusses the results obtained in the laboratory. Finally, "Conclusion" concludes the findings obtained dynamic emulation for the SWWEC.

**Fig. 1:**
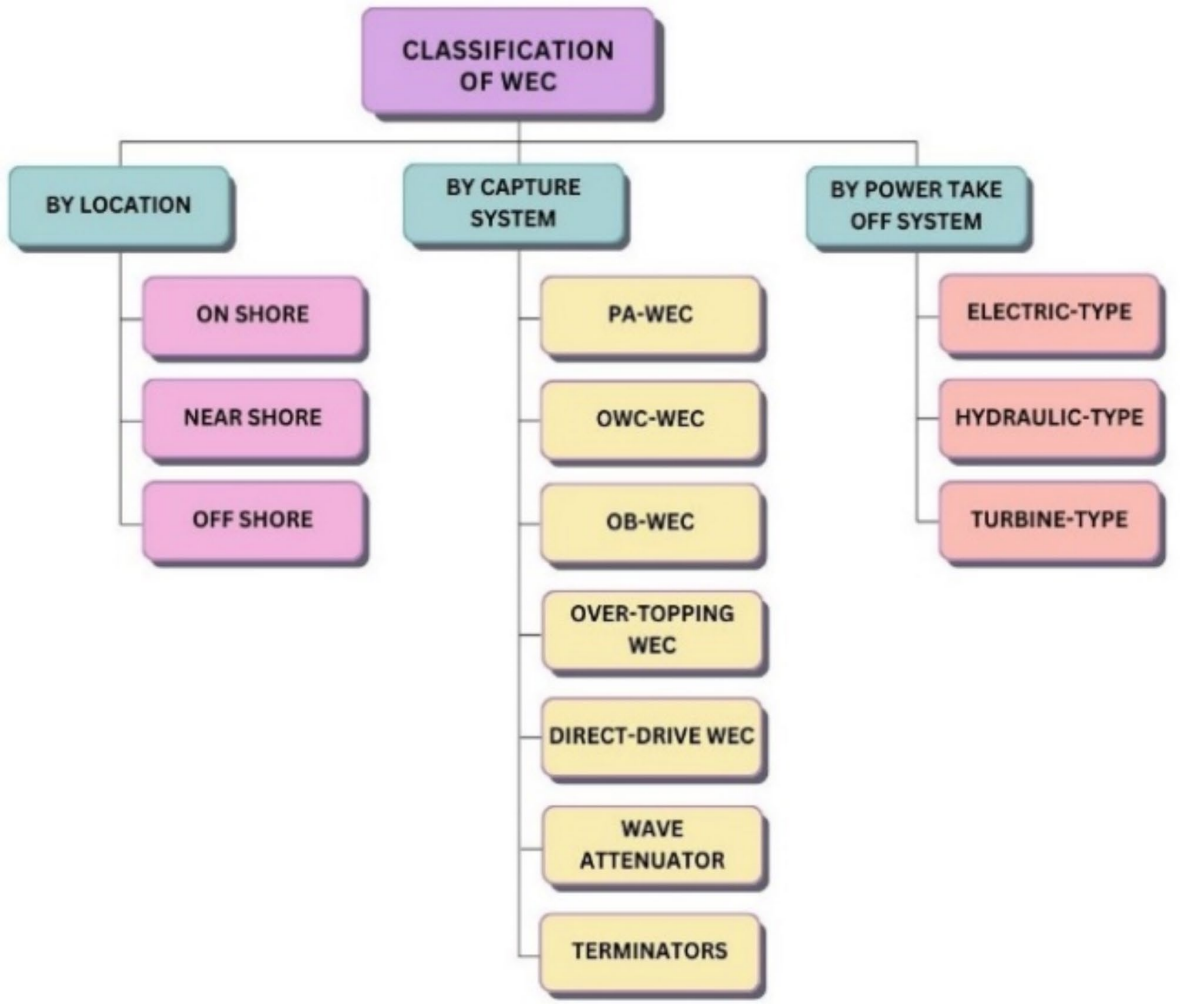
Classification of the WEC based by Location, Capture System and Power Take Off System.

**Fig. 2:**
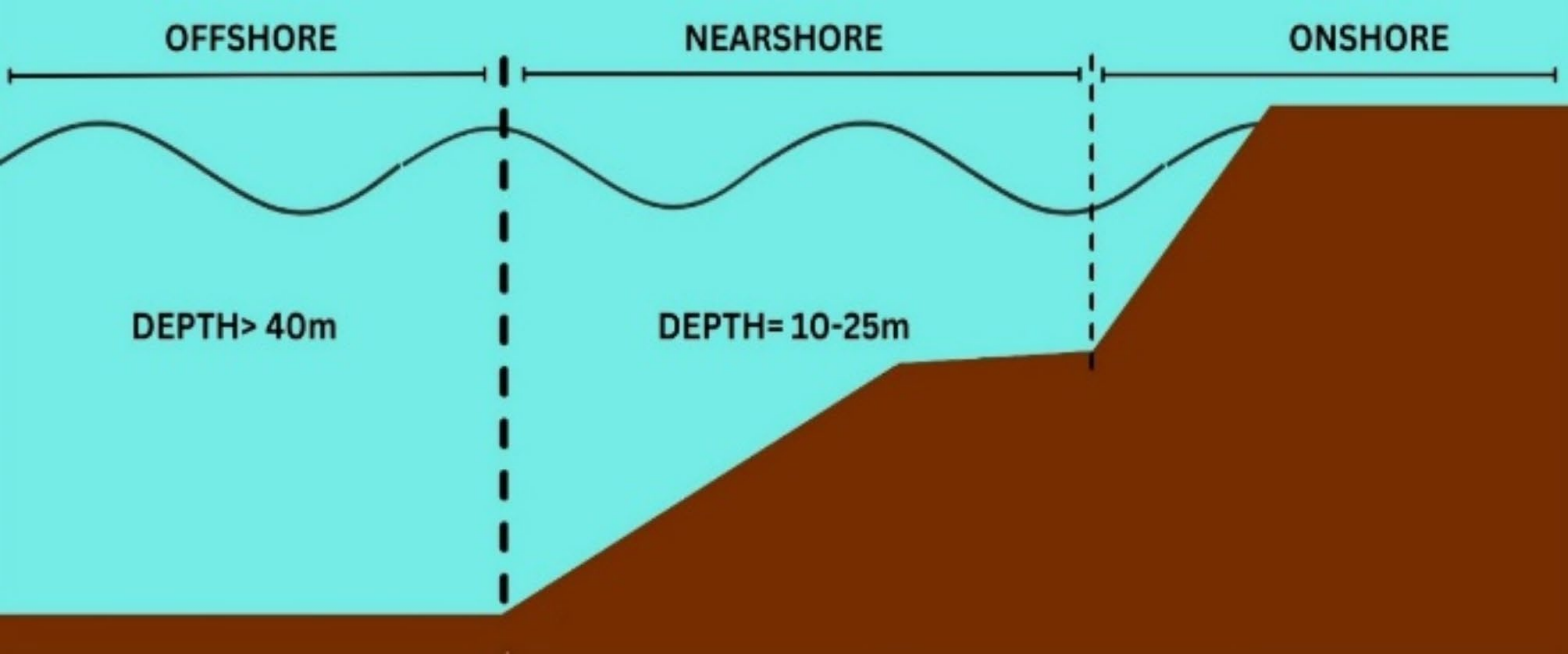
Schematic representation of the locations of Onshore, Nearshore and Offshore.

Table 1 shows different types of WECs based by location, capture system and power take off system are shown in Fig. 1. The location of the deployed WEC determines the capture system and power take off system accordingly, Fig. 2 shows the classifications of the location.

**Table 1:** List of full-scale installed WECs across the globe.

Name reference	Installed capacity	Category	Country	Grid-connected
Aqua buoy^31^	250 kW	PAWEC	Ireland-Canada-Scotland	Yes
Powerbuoy^32^	150 kW	OBWEC	America	No
WaveStar^33^	6 MW		Denmark	Yes
Pelamis^34^	750 kW		England	Yes
Wave Dragon^35^	12 MW	Overtopping WEC	Denmark	Yes
Penguin^36^	0.5–1 MW	Direct-drive WEC	Finland	Yes
Ocean energy buoy^37^	1.25 MW	OWCWEC	Ireland	No
GreenWave Oceanlinx^38^	450 kW/1 MW		Australia	Yes

J.C.C. Henriques et al.^6^ proposed a design of oscillating-water-column WEC with an application to self-powered sensor buoys. Yung-Lien Wang performed a numerical study on the optimal size of the cylindrical buoy based on the wave characteristics^7^. Centre for renewable electric energy conversion, Uppsala University proposed and implemented the concept of WECs where the concept of buoy on sea surface connected to linear generator placed on the seabed^8^. Northwest National Marine Renewable Energy Centre reported the development of Ocean Sentinel instrumentation buoy which is actually a surface buoy as well as oceanographic meteorological automatic device for the testing of immature WECs at the site of ocean^9^. Marine energy harvesting is currently exploring various aspects, and research has classified different WECs including Pelamis, Aquabuoy, Wave Dragon, and Oscillating Water Column. Modesto Amundarain, Mikel Alberdi have reported and explained clearly about the control scheme implemented for the generation of energy through the oscillating water column^10^. Feng Wu, Xiao-Ping Zhang mentioned about the development of AWS based WEC system where the generator and grid side converter control scheme are emphasized^11^. WECs on the basis of hydrodynamic principle based on point absorber principle is introduced by Budal and Falnes^12^. Nicolas Müller and Samir Kouro mentioned about the wave dragon principle for the generation of electric energy which consists of several low voltage source converters with back-to-back configuration^13^. Offshore WECs necessitate sea cables to connect to the onshore grid. Enhancing transmission and cost efficiency, a seabed-based offshore marine substation connects all WECs. Uppsala University developed and implemented this technology^8^. Some researchers suggest integrating wind and wave farms with a shared offshore substation to enhance substation efficiency on the seabed. Cost and expenditure aspects for wave energy farm installations are explored by Fergus Sharkey and Elva Bannon in^14^. Hui Huang et.al implemented different control schemes for those converters for proper control. This control schemes can be applied to wave energy conversion system^15^ as the controller scheme is very much essential for converters present in the WECs, Adel A. A. Elgammal proposed adaptive Fuzzy Logic Sliding Mode Controller for grid side converter control^16^. Inspite of different proposed simulation, M. Rahm et.al implemented and tested the marine substation which is clearly seen in^17^. 

Table 2 shows a list of researchers with their objectives and contributions in developing novel WECs for harnessing clean wave energy.

**Table 2:** WEC researcher insights: objectives & contributions.

Type of WEC	Ref	Objectives	Remarks
Point absorber wave energy converter (PAWEC)	^39^	To employ a turbine generator propelled by the Magnus effect in a point-absorber WEC	Simulation model of the proposed design is developed, and the results are presented
	^40^	To develop and numerically analyze a point-absorber with 2 DOF using CFD	The heaving and pitching motions can be considered interdependent, simplifying the wave energy conversion to the combined single DOF motion of heave and pitch
	^41^	To examine the implementation of a backward bent duct buoy (BBDB) with a point absorber (PA)	The results suggest optimizing hybrid WEC designs and indicate potential synergy between BBDB and PA for efficient use of ocean space for energy
	^42^	This research demonstrates the simulation of harnessing energy using WEC from ocean waves in Indonesia	The average value of electrical powers generated during the simulation and accumulated electrical powers were presented
	^43^	To numerically analyze a self-reacting WEC	Numerical analysis has been conducted using both frequency-domain and time-domain approaches for regular waves
Swing motion bouy	^4^	To check the performance of a longitudinal swing motion of the buoy	The results obtained after the numerical analysis show that the performance of the buoy with swing motion has improved when compared to heave motion
Oscillating-array-buoys	^5^	To optimize the energy capturing mechanism of the oscillating array-buoys	The experimental values show that there is an improvement of 38% when compared to the basic model
	^44^	The oscillating array of buoys is integrated with the development of a semi-submersible platform	The numerical analysis of the test rig shows a combined average of 18.115% in regular and irregular waves
Oscillating water column wave energy converter (OWCWEC)	^6^	To design two self-powered sensor buoys for long term monitoring based on the oscillating-water-column principle	The performance of the designed buoys was analyzed, and the results shown give positive feedback for implementing them in the future applications
	^45^	To design and model 2 different types of OWC-WEC	The results obtained from the hydrodynamic performance, mooring tension results and free-decay tests are presented and discussed
Sealed-buoy wave energy converter (SBWEC)	^46^	To capture energy from low energy flow density sea areas using a SBWEC	The estimated efficiencies of the SBWEC calculated were 54.44%

Performance enhancements of WEC is also a major criterion for energy harnessing from wave energy resources therefore various researchers have proposed efficient control techniques. In the present era to enhance the control of efficiency adaptive controllers , robust controller and intelligent controllers are proposed and implemented for various categories of renewable energy system controller such as PV systems, wind systems and wave energy systems**.***Nagwa F. Ibrahim *et al.^18^ proposed an effective PI controller for a dynamic voltage restorer to reduce the PQ problems, using ARO to achieve optimal tuning. *Mohamed Metwally Mahmoud *et al.^19^ presented the application of a whale optimization algorithm based on a fractional order proportional-integral controller for unified PQ conditioner and STATCOM tools. *B. Srikanth Goud *et al*.*^20^ aimed to enhance the PQ of the electronic equipments for the consumers by implementing a dynamic voltage restorer. *Mohamed Metwally Mahmoud*^21^ implemented a WHO method to optimally design a wind turbine system based on PMSG. *Mohamed Metwally Mahmoud *et al.^22^ proposed a Harris Hawks algorithm to improve the dynamic voltage resistor’s control system for enhancing the VQ in low levels of voltages in smart homes. *Nagwa F. Ibrahim *et al.^23^ developed two MPPT algorithms: ANN and CS, to investigate the PQ of the electricity harnessed from renewable energy resources which include DC-DC, DC-AC converters, PV, power grid, filter and control schemes. *Santanu Kumar Dash and Pravat Kumar Ray*^24^ developed a new version of JAYA algorithm which has two distinct objective functions to optimally control the PV-UPQC. From the above literatures controller like adaptive control, optimization based control, and robust control increase the efficiency of renewable energy based systems. Therefore, these control methodologies can be applied to WECs. Against this background, the aim of this research are as follows:

- Instantaneous control theory dq-method has been implemented for the control of both grid and generator side controller. This method is effectively used to control the variable voltage and frequency generated by the variance of the waves.
- A novel efficient 3D CAD design integrating three sources of renewable energy i.e. solar, wind and wave energy has been presented.
- Dynamic emulation of the SWWEC in real-time simulation environment by driving the DC generator using an electrical motor has been achieved.

In this article, "Wave Concept and Theory for floating buoy:" illustrates the theory behind the wave concept and characteristics of the waves in the ocean. "Case 1: wave energy converter of a floating buoy Archimedes Wave Swing:" explains the design and control of the conventional PAWEC. "Case 2: design of wave energy converter" proposes a new design of the point absorber WEC in which the electrical generator is kept above the surface of water instead of completely keeping it submerged in a water-tight container. "Design of the buoy of WEC" discusses the design of the buoy of WEC. The key advantages of the proposed SWWEC are listed in "Advantages over a point absorber WEC". "Laboratory validation and result analysis" discusses the results obtained in the laboratory. Finally, "Conclusion" concludes the findings obtained dynamic emulation for the SWWEC.

## Wave concept and theory for floating buoy

The utility factor is important for any schemes proposed and is the key parameter in the economics of renewable energy production. This utility factor can be defined as

1 $$\alpha =\frac{{P}_{a\mathit{var}age}}{{P}_{rated}}=\frac{W}{{P}_{rated}.8760}$$

where the rated power is denoted by *P*_*rated,*_ and average power is denoted as* P*_*average*_. The investment payback is determined by the annually produced energy *W.* The comparison of the utility factors for wind, solar and wave energy can be obtained through Eq. (1) which says that the wave energy has the higher utility factor. The generated energy flux by sea waves attenuates on a slower time scale in comparison to wind and solar. Due to the higher utility factor claimed by the wave energy^28,29^, it is required to be considered for the design of the wave energy farms.

The wave theory presents the energy in the wave as potential energy and kinetic energy. The time averaged wave power per unit width L of the wave front is given by

2 $$\frac{dP}{dL}=cT{H}^{2}$$

where *c* is approximately equal to 976 Wm^-3^ s^-1^ the height *H* of the wave is twice the amplitude means *H* = *2A.*

### Wave theory

The statistical characteristic of ocean waves can be modelled through vast number of parameters where the irregular waves are described by a spectrum *S*_*f*_ indicates the amount of wave energy at different wave frequencies *f*. The spectral characteristic and the wave parameters can be calculated using the time series representation with spectral moment *m*_*n.*_ The area under the spectral energy function is denoted as *m*_*0.*_ For the calculation of higher spectral can be calculated by

3 $${m}_{n}={\int }_{0}^{\infty }{f}^{n}S(f)df$$

In this equation the *n* can take any integer value both positive and negative. Some of the important wave parameters are as follows:

(i) Standard deviation of the sea level can be denoted as the significant wave weight *Hs*, corresponding to the significant wave height estimated from the spectral moments *H*_*m0*_.

$${H}_{s}\cong {H}_{m0}=4\sqrt{{m}_{0}}$$

(ii) *T*_*e*_ is the energy period or mean wave period with respect to the spectral distribution of energy *T*_*-10*_, is defined by

$${T}_{e}={T}_{-10}=\frac{{m}_{-1}}{{m}_{0}}$$

The zero-up crossing period* T*_*z*_ and the peak wave period* T*_*p*_ are the most important wave parameters for the wave period.* T*_*p*_ is the predominant wave period and *T*_*e*_ represents the average energy period of the wave spectrum.

For example, Atlantic oceanic values of *T*_*e*_ and *H*_*s*_ value is between 5 and 15 s and 0 and 10 m respectively. On the basis of these parameters, the omnidirectional wave power (kW/m) can be defined with the wave number based on the energy period k_e_ and taking the water depth *h* into consideration, by

4 $${P}_{wave}=\frac{\rho {g}^{2}}{64\pi }{H}_{m0}^{2}{T}_{e}\left[1+\frac{2{k}_{e}h}{\mathit{sinh}2{k}_{e}h}\right]\mathit{tanh}{k}_{e}h$$

### Wave characteristics

**Fig. 3:**
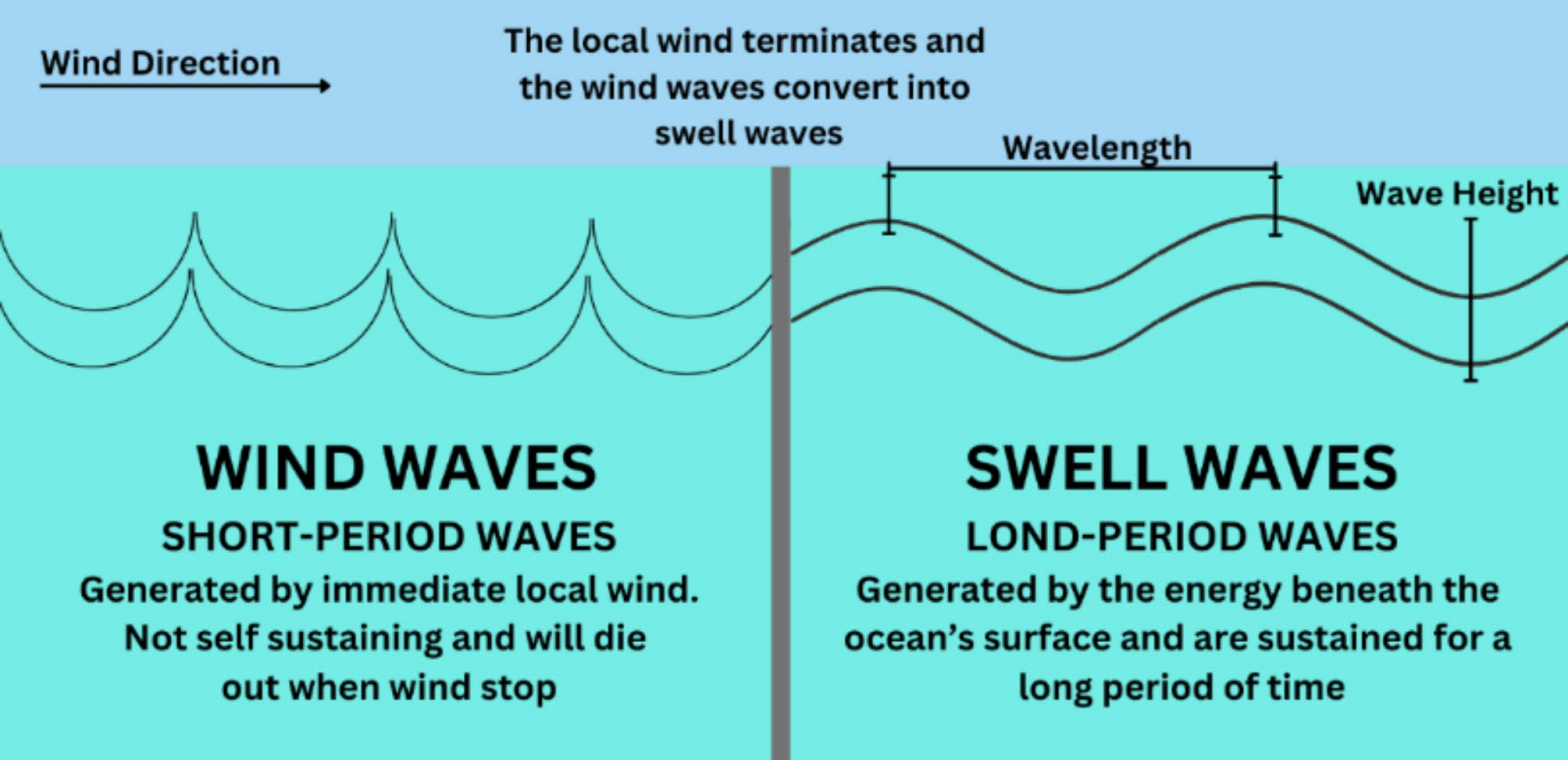
Difference between Short-Period Waves and Long-Period Waves.

In the ocean, waves are typically categorized into two primary types: long-period swell waves and short-period wind waves Fig. 3. Swell waves, characterized by their extended wavelengths and gradual crests, constitute the primary source for wave energy conversion. Conversely, the wind waves or short-period waves, with their shorter wavelengths and abrupt crests, tend to induce undesirable energy rippling and should thus be minimized in design considerations.

Fundamentally, waves in ocean transmission are characterized by three critical parameters: wavelength, which denotes the distance between consecutive crests or troughs; wave amplitude, representing the height of a wave from its trough to its crest; and wave velocity, signifying the speed at which a wave propagates through the water medium. These factors collectively influence the behaviour and impact of waves on the design of buoy for the WEC.

## Case 1: wave energy converter of a floating buoy Archimedes wave swing

**Fig. 4:**
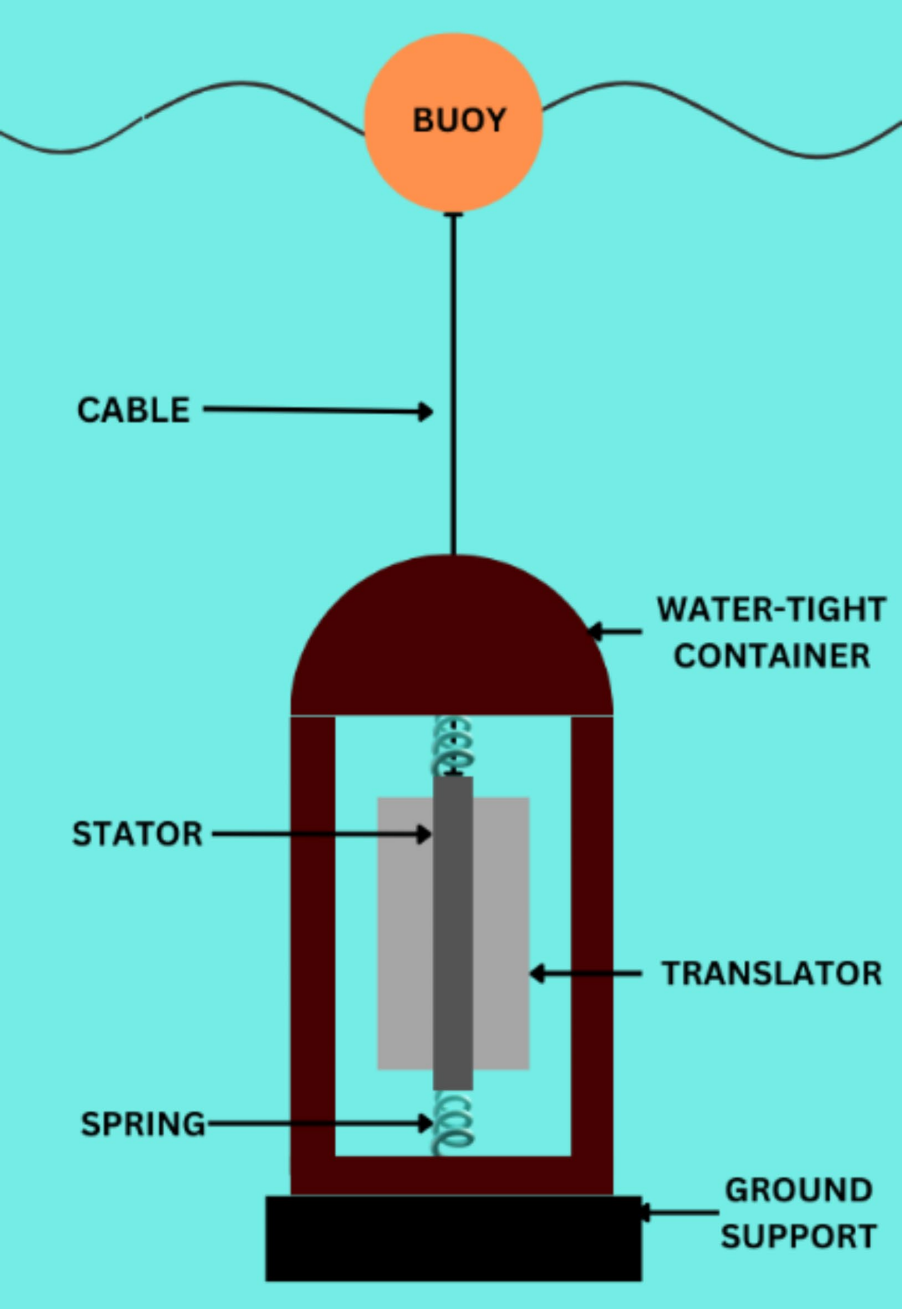
Conventional Design of PAWEC system.

There are several types of WECs proposed and under developmental condition for the harnessing of the wave energy, however the PAWEC is the most popular one. Figure 4 shows the schematic diagram of a conventional PAWEC system. There are different forces acting on it when it is on the sea surface. The movement of the buoy depends on the waves passing through it; the buoy is lifted, and the corresponding motion is reflected with the translator in the generator^29^. The relative motion in between the stator and magnets generates the voltage in the stator windings^25^.

The piston present in the generator is driven by the buoy with a force corresponding to the lift force where the diameter is much smaller than the wavelength. The actual motion of the piston connected to the buoy is as follows:

5 $$m\frac{{d}^{2}x}{d{t}^{2}}={F}_{buoy}+{F}_{spring}+{F}_{em}$$

The present equation shows that the total force is a sum of buoyant force, mechanical spring force and generator inductive force.

6 $${F}_{spring}=-{F}_{0}-{k}_{sp}x$$

7 $${F}_{em}=-{k}_{em}\frac{dx}{dt}$$

For electromagnetic inductive force generation, the computation of the retarding effect on the piston associated to stator currents and load applied. The AWS tolerates damping and energizing motion of the translator.

8 $$mx={F}_{s}+{F}_{b}+{F}_{em}+{F}_{es}+mg$$

In the denoted equation for WECs, m is the mass of the translator, x is the acceleration*. F*_*s*_ and *F*_*b*_ are the spring force and buoy force respectively. The end stop force at the top is denoted as *F*_*es*_ and* F*_*em*_ is the electromagnetic force depending on electrical damping of the generator. The relation between the electromagnetic force *F*_*em*_ and the damping function $\gamma$ can be represented as follows:

9 $${F}_{em}=\gamma {A}_{fac}x$$

where the active area of stator is represented as A_fac_ and the required operating range of active area of stator is given as $0\le\upgamma \le 1$.

The damping function can be obtained by the relation of the force and the velocity represented here as

10 $$\gamma =\frac{{P}_{abs}}{{A}_{fac}{x}^{2}}$$

where the *P*_*abs*_ is represented for absorbed power.

11 $${P}_{abs}={P}_{mech}+{P}_{load}+{P}_{cu}+{P}_{iron}\underset{\_}{\sim }{P}_{load}+{P}_{cu}$$

*P*_*iron*_*, P*_*mech*_*, P*_*cu*_ are the iron losses, mechanical losses, and copper losses respectively. The copper losses are the conductor losses in armature winding and the sea cable.

**Fig. 5:**
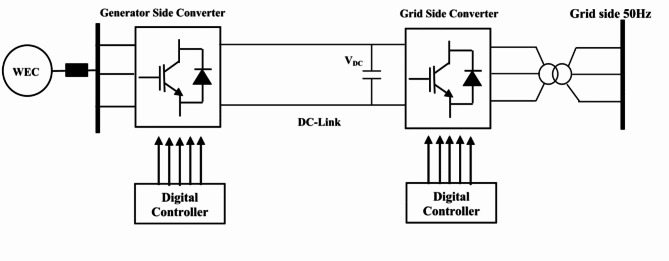
Configuration of the power circuit and controller for the WEC.

**Fig. 6:**
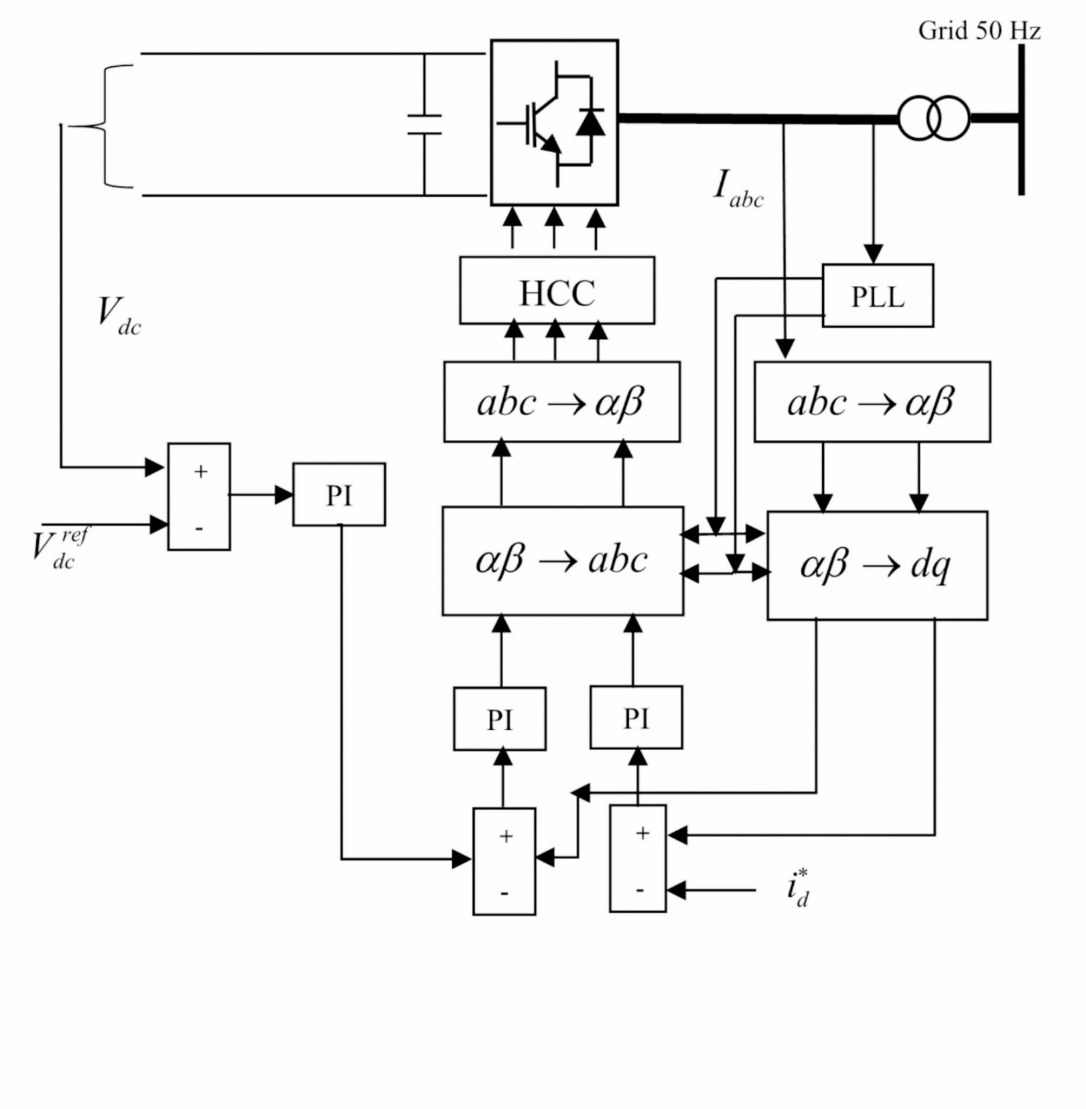
Grid side controller design of PAWEC.

Figure 5 shows the configuration of the total system of the power circuit and controller required for converting power from WEC to the utility grid. The AC/DC converter in the configuration is designed to convert the variable frequency generated by the AC power for the regulation of the DC power. The controlled and regulated DC power can charge battery storage to supply the DC loads. The DC power is required to deliver to the DC side of the 3-phase inverter to be converted to the 3-phase AC power for the AC loads and the grid. Problems arise due to the variation of the terminal DC voltage for the variation of the wave speed and the frequent change in load. To overcome this problem an effective method of control is required for the control of the DC bus voltage. Instantaneous control theory dq-method has been used in the present paper as given in Fig. 6 for the control of both grid and generator side controller. This method is effectively used to control the variable voltage and frequency generated by the variance of the waves.

## Case 2: design of wave energy converter

**Fig. 7:**
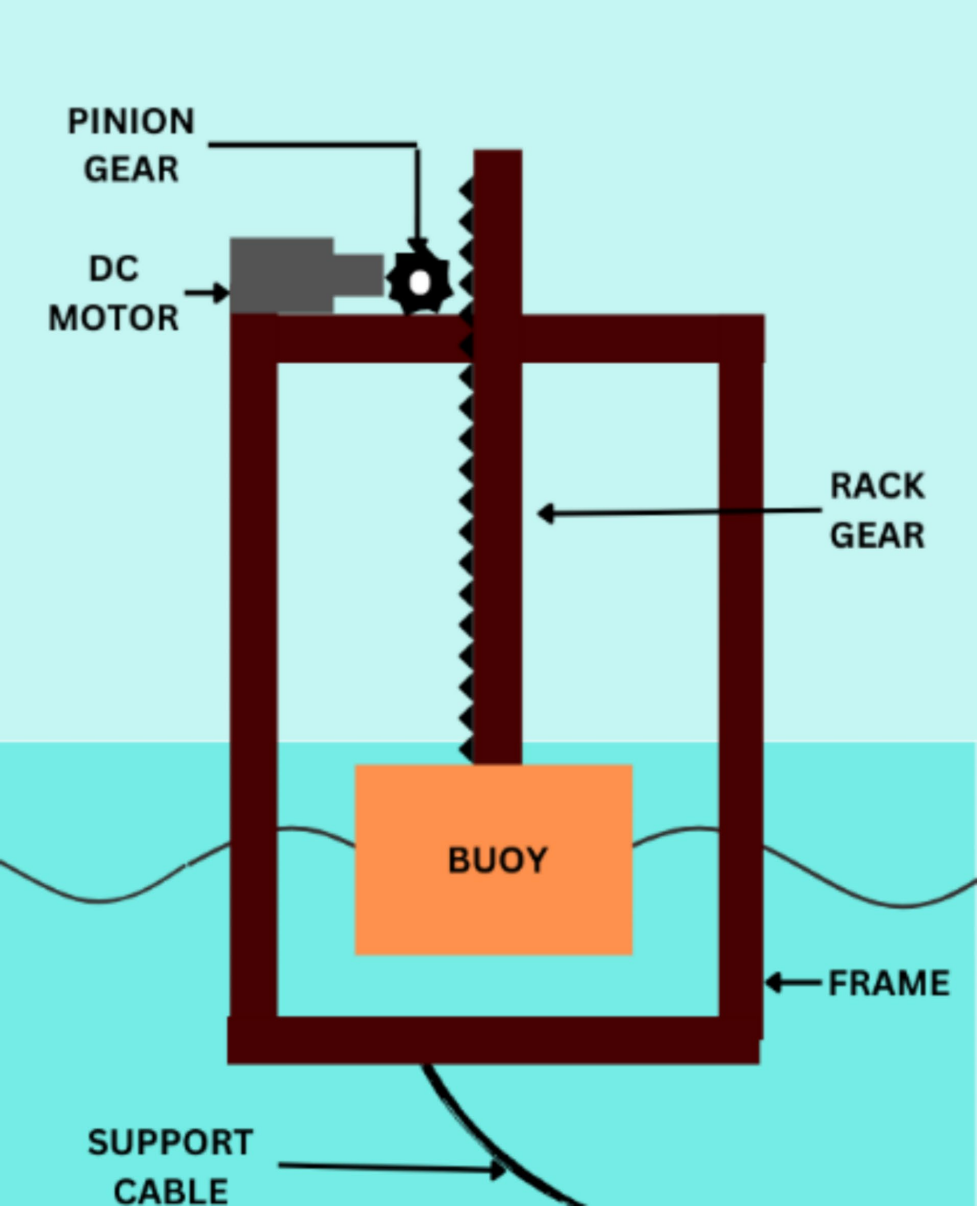
Proposed Design of PAWEC.

The SWWEC design proposed in this paper is a modification of the PAWEC design (Fig. 7) where instead of keeping the main body consisting of the generator and electronic components such as batteries underwater in a seal proof container, we have directly kept it above the floating buoy which completely eliminates the increased cost of manufacturing a watertight container and the installation costs inside the ocean.

**Fig. 8:**
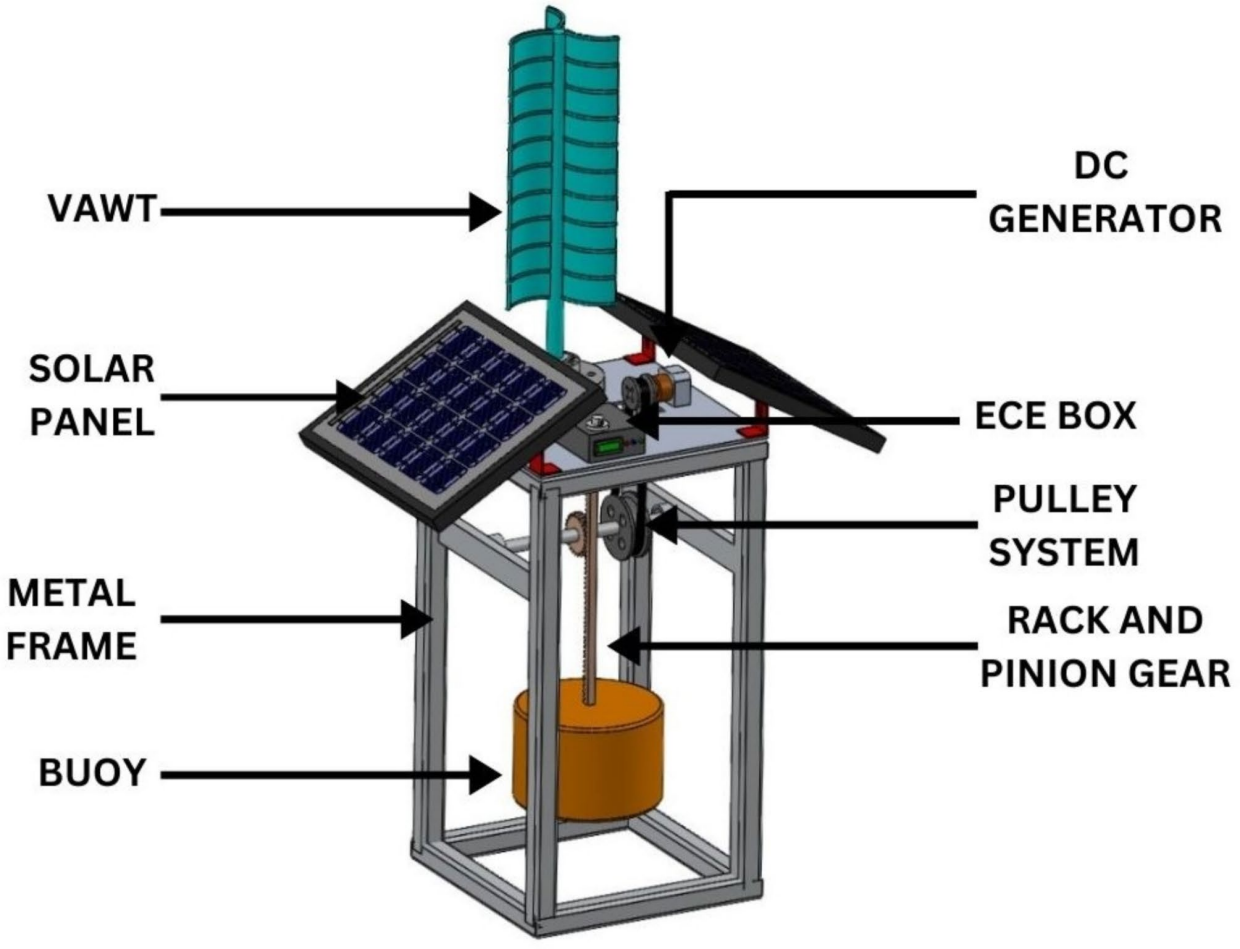
Conceptual design of the proposed hybrid SWWEC.

**Fig. 9:**
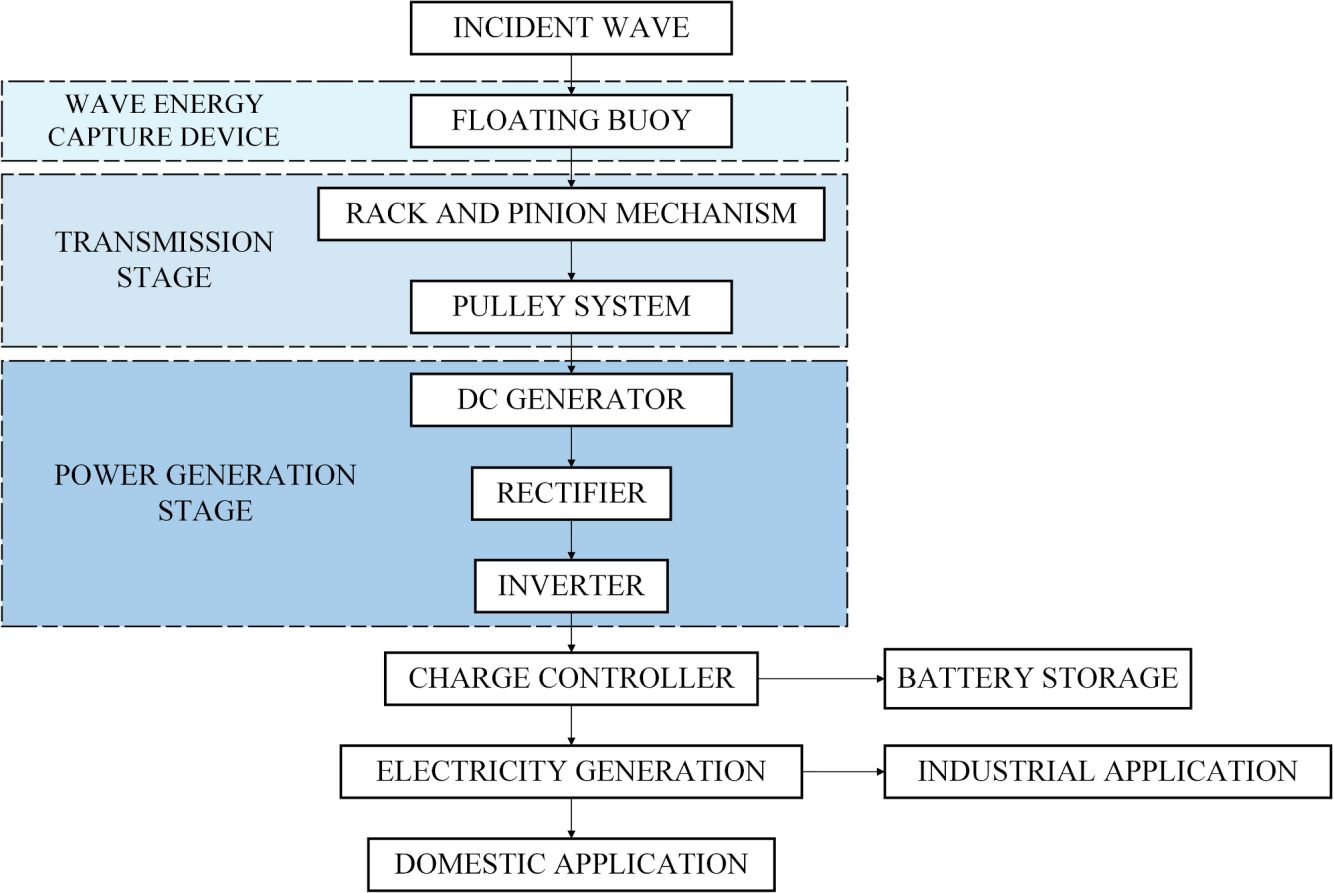
Wave Energy flow diagram in the proposed hybrid SWWEC.

Table 3 shows the front view, side view, top view, and isometric view of the proposed hybrid SWWEC. The SWWEC outlined in this paper (Fig. 8) comprises several key components: a sturdy metal frame, PV panels, VAWT, cylindrical floating buoy, rack and pinion gear mechanism, pulley system, DC generator, and a controller^30^. The frame serves as the backbone of the system, offering structural support and stability to the other components. It ensures that the various parts of the SWWEC are securely held together and properly positioned for efficient energy conversion. Monocrystalline PV panels were chosen for this application due to their higher efficiency, increased longevity and better durability than the polycrystalline PV panels. The PV panels are situated on top of the SWWEC for maximum absorption of the global radiation received by the sun, it also serves as a shelter for the electronic components which reduces the heat gained directly from the sun’s radiation avoiding frequent replacements of damaged electronic components. The VAWT is also situated on top of the SWWEC for maximum capture of wind energy. The design of the 3-bladed VAWT is inspired from the savonius VAWT due to its simple construction, reduced noise, minimal wear and the ability to work in low wind speed conditions. The wave energy flow diagram of the proposed SWWEC is shown in Fig. 9. The cylindrical floating buoy performs a heaving motion when a sea wave passes through. Simultaneously the rack also heaves in the upward direction causing the pinion to rotate. This rotation of the pinion also rotates the rod to which it mounted on, then the larger pulley also rotates with the rotation of the rod and transmits the rotation to the smaller pulley via a belt. The smaller pulley acts as an input shaft for the DC generator. A pulley system with a ratio of 1:2 is considered where for each rotation of the larger pulley results in two rotations of the smaller pulley which also results in the increased input rotation of the DC generator, hence increasing the power generated. The DC generator produces power, which is subsequently rectified by a full-wave rectifier. This rectified power then flows through an inverter, followed by a charge controller, and finally stored in the battery storage unit. The entire SWWEC system is moored to the seabed to prevent it from floating away from the location at which it is installed. The power captured by solar, wind and wave are stored in the battery which can be utilized for either domestic or industrial use according to the location of the SWWEC.

**Table 3:**
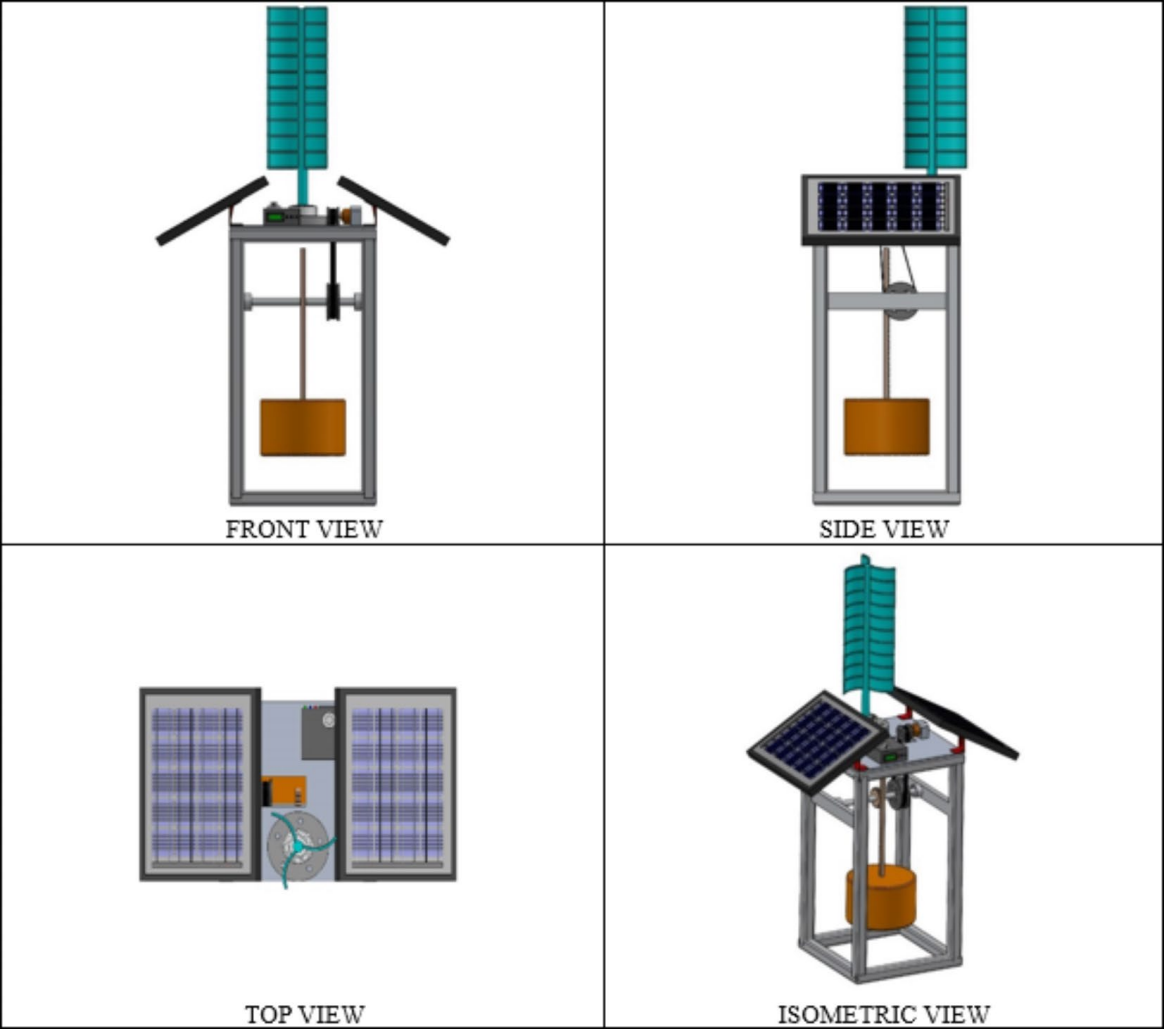
Front view, side view, top view & isometric view of the proposed hybrid SWWEC.

### Forces acting on a WEC

**Fig. 10:**
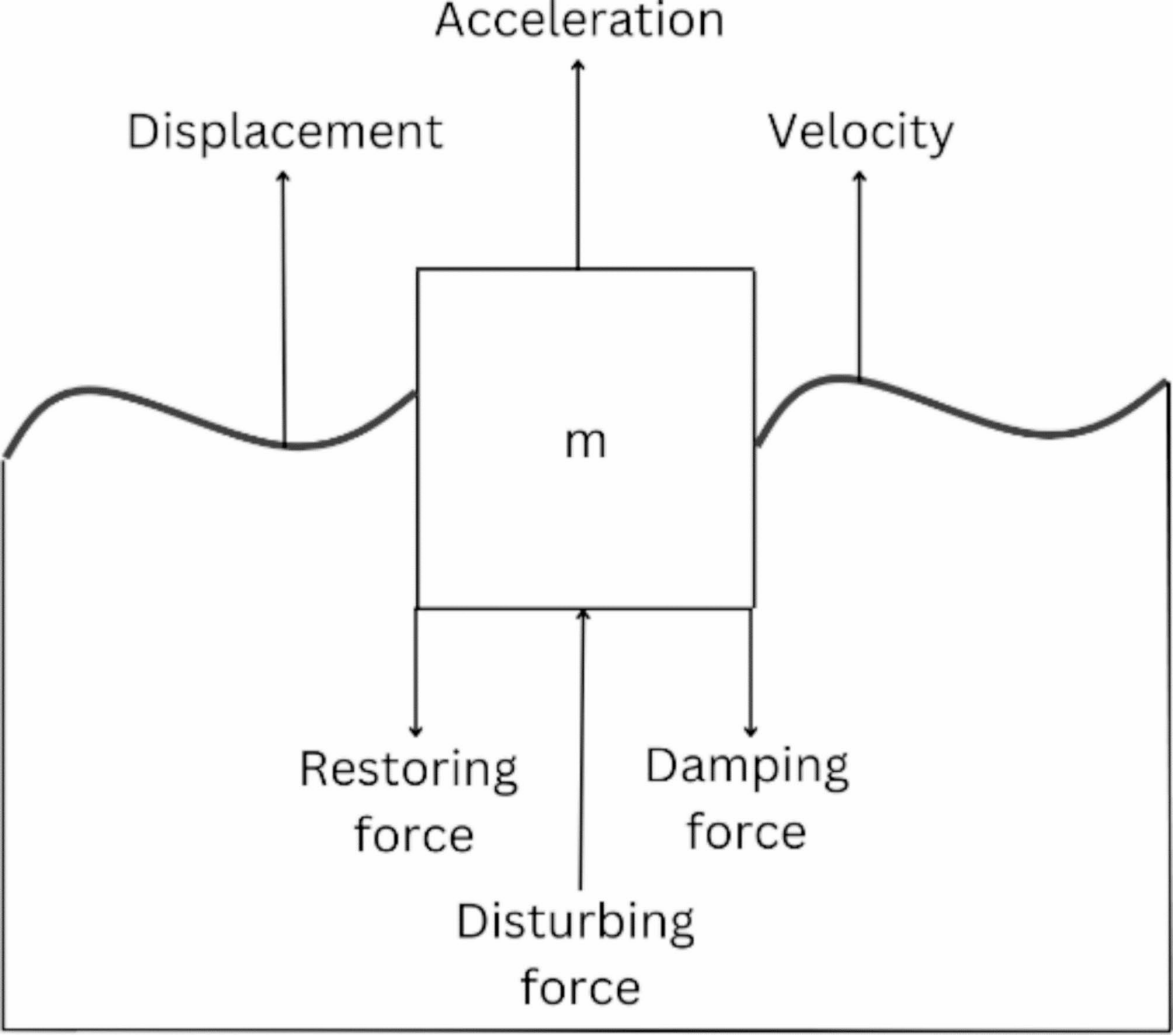
Forces acting on a WEC.

The motion of the WEC depends on the amplitude and angular frequency of the incident wave. The forces acting on a WEC has been presented in Fig. 10. This produces a periodic disturbing force $Fcos\left(\omega t\right)$ which is controlled by a restoring force, produced by changing buoyancy and a damping force caused by the friction, energy extraction and radiation^26^.

12 $$\underbrace {Fcos\left( {\omega t} \right)}_{Applied\;force} - \underbrace {D\dot y}_{Damping\;force} - \underbrace {Sy}_{Restoring\;force} = \underbrace {m\ddot y}_{mass\;{\text{X }}acceleration}$$

Hence.

13 $$m\ddot{y}+D\dot{y}+Sy=Fcos\left(\omega t\right)\text{ or }Re. F \text{exp}(i\omega t)$$

To simplify the problem considering the natural undamped oscillation so that $D=F=0.$

Thus,

14 $$m\ddot{y}+Sy=0$$

Substituting the value of $y=A \text{exp}(i{\omega }_{o}t)$ in Eq. (13)

$$mA{(i{\omega }_{o})}^{2} \text{exp}(i{\omega }_{o}t)+SA \text{exp}(i{\omega }_{o}t)=0$$ or $$-m{{\omega }_{o}}^{2}+S=0$$

15 $$=> {\omega }_{o}=\sqrt{\frac{S}{m}} \quad ({\rm where}\, {\omega }_{o} \, {\rm \, is \, the \,resonant \, angular \, frequency})$$

Now reintroducing the damping term $D\dot{y}$ in Eq. (14)

16 $$m\ddot{y}+D\dot{y}+Sy=0$$

Now substituting the form $y=A \text{exp}(\alpha t)$ and its differentials and cancelling $A \text{exp}(\alpha t)$ throughout

17 $$m{\alpha }^{2}+D\alpha +S=0$$

18 $$\alpha =\frac{-D\pm \sqrt{{D}^{2}-4mS}}{2m}$$

Unless

${D}^{2}-4mS<0$, $\alpha$ is real and negative and the displacement decreases to zero asymptomatically with time without periodic motion, which is not relevant to wave energy devices and so the solution of interest is

19 $$y=\text{exp}\left(-\frac{Dt}{2m}\right)[{A}_{1}\text{exp}\left(i{\omega }_{d}t\right)+{A}_{2}\text{exp}\left(-i{\omega }_{d}t\right)]$$

where,

20 $${\omega }_{d}=\sqrt{\frac{S}{m}-\frac{{D}^{2}}{4{m}^{2}}}$$

Putting, ${A}_{1}=\frac{A\left(cos\delta +sin\delta \right)}{2}$ and ${A}_{2}=\frac{A\left(cos\delta -sin\delta \right)}{2}$ in Eq. (8) we get

$$y=\text{exp}\left(-\frac{Dt}{2m}\right)A[\frac{\left(cos\delta +sin\delta \right)\text{exp}\left(i{\omega }_{d}t\right)}{2}+\frac{\left(cos\delta -sin\delta \right)\text{exp}\left(-i{\omega }_{d}t\right)}{2}]$$

21 $$y=\text{exp}\left(-\frac{Dt}{2m}\right)\text{cos}({\omega }_{d}t-\delta )$$

Now to satisfy the RHS of Eq. (13) the solution for the disturbing frequency should be of the form,

22 $$y=\text{B exp}(i\omega t)$$

Substituting y and its derivatives in Eq. (13)

23 $$-m{\omega }^{2}B+iD\omega B+SB=F$$

24 $$=>B=\frac{F}{\left(S-m{\omega }^{2}\right)+iD\omega }$$

Now using the properties of complex numbers, the denominator can be written as

25 $$\{\sqrt{{\left(S-m{\omega }^{2}\right)}^{2}+{D}^{2}{\omega }^{2}}\}\text{exp}(i\alpha )$$

where,

26 $$\alpha ={\text{tan}}^{-1}\{\frac{D\omega }{S-m{\omega }^{2}}\}$$

So that if $F$ is complex $F=|F|\text{exp}(i\theta )$ and

27 $$y=\frac{|F|\text{exp}[i\left(\omega t+\epsilon \right)]}{\sqrt{{\left(S-m{\omega }^{2}\right)}^{2}+{D}^{2}{\omega }^{2}}}$$

where $$\epsilon =(\theta -\alpha )$$.

### Equations on the cylindrical buoy

Considering the horizontal cross-section of a cylindrical buoy,

$$A=\pi {a}^{2}$$

For a vertical displacement y, the change in buoyancy force is $\rho g(Ay)$, hence the restoring force coefficient is,

28 $$S=\rho gA=\rho g\pi {a}^{2}$$

Now the damped natural frequency ${\omega }_{d}$ by observing in the period $T={t}_{3}-{t}_{1}$ is

29 $${\omega }_{d}=2\pi /{t}_{3}-{t}_{1}$$

Also, $${\omega }_{d}=\sqrt{\frac{S}{m}-\frac{{D}^{2}}{4{m}^{2}}}$$ and $$y=A\text{exp}\left(-\frac{Dt}{2m}\right)\text{cos}({\omega }_{d}t-\delta )$$

30 $$\frac{{y}_{1}}{{y}_{3}}=\frac{\text{exp}\left(-\frac{D{t}_{1}}{2m}\right)}{\text{exp}\left(-\frac{D{t}_{3}}{2m}\right)}=\text{exp}(\frac{2\pi D}{2m{\omega }_{d}})$$

For convenience, introducing a damping ratio $\Delta$ which is defined as the ratio of actual damping coefficient $D$ to the critical damping coefficient when ${\omega }_{d}=0$ and $D=\sqrt{2mS}$. Hence,

31 $$\Delta =\frac{D}{\sqrt{2mS}}=\frac{D}{2m{\omega }_{o}}$$

From Eqs. (30) and (31)

32 $$\frac{{y}_{1}}{{y}_{3}}=\text{exp}(\frac{2\pi \Delta {\omega }_{o}}{{\omega }_{d}})$$

Now, (20) can be written as

33 $${\omega }_{d}={\omega }_{o}\sqrt{1-{\Delta }^{2}}$$

And (32) becomes,

34 $$\frac{{y}_{1}}{{y}_{3}}=\text{exp}(\frac{2\pi \Delta }{\sqrt{1-{\Delta }^{2}}})$$

Rearranging the above equation

35 $$\Delta =\frac{\text{ln}(\frac{{y}_{1}}{{y}_{3}})}{\sqrt{4{\pi }^{2}+({\text{ln}\frac{{y}_{1}}{{y}_{3}})}^{2}}}$$

The instantaneous damping force due to energy extraction $={D}_{e}\dot{y}$ so that the instantaneous energy extraction rate ${=D}_{e}{\dot{y}}^{2}$,

36 $$\text{We know that }y=Ccos(\omega t+\epsilon )$$

37 $$\text{Where }C=\left|F\right|/\sqrt{{(S-m{\omega }^{2})}^{2}+{D}^{2}{\omega }^{2}}$$

38 $$D={D}_{e}+ {D}_{f}+{D}_{r}={D}_{e}+{D}_{L}$$

Differentiating (36)

39 $$\dot{y}=-C\omega sin(\omega t+\epsilon )$$

Therefore, the instantaneous energy extraction rate of power

40 $${=D}_{e}{\dot{y}}^{2}={D}_{e}{C}^{2}{\omega }^{2}{sin}^{2}(\omega t+\epsilon )$$

41 $$\text{Mean power }=\overline{{D }_{e}{\dot{y}}^{2}}={D}_{e}{C}^{2}{\omega }^{2}\overline{{sin }^{2}(\omega t+\epsilon )}$$

Now the mean square of a sine wave over a period of ½, hence the mean power is,

42 $$\overline{P }= {D}_{e}{C}^{2}{\omega }^{2}/2\text{ W}$$

The maximum power demand when the undamped natural frequency be matched to the forcing frequency of the waves and the ${D}_{e}={D}_{L}+ {D}_{f}+{D}_{r}$,

43 $${\overline{P} }_{max}={|F|}^{2}/8{D}_{L}\text{ W}$$

## Design of the buoy of WEC

The motion of the buoy considered in this paper is a heave type of motion. The wave velocity decides the decay time as the buoy will not be able to capture the wave energy if the decay time is too long. Hence, when creating a buoy’s response to big, slow waves, it’s best for the buoy to move smoothly up and down. This helps stop the waves from causing too much bouncing or splashing around.

A WEC buoy’s performance is influenced by three key factors discussed in wave characteristics. The buoy operates like a particle, undulating with incoming waves as long as the wavelength isn’t excessively short. Typically, the size of the buoy is kept below one-tenth of the wavelength. If the wavelength is too short or matches the size of the buoy, the buoy may straddle the wave’s crest and trough simultaneously, resulting in inefficient energy capture.

The amplitude of the wave is crucial as it determines the vertical potential energy of the buoy and the amount of energy captured. Wave velocity, representing the speed of wave transfer, impacts the dynamic response of the buoy. A slow buoy dynamic response in fast wave velocity conditions leads to the wave passing the buoy without significant motion. Conversely, a fast buoy dynamic response in slower wave velocity conditions reduces the captured wave energy as mentioned by Yung-Lien Wang^7^.

## Advantages over a point absorber WEC

The design of the hybrid SWWEC outlined in this paper offers several notable advantages over the conventional Point Absorber Wave Energy Converters (PAWECs) as shown in Table 4:

**Table 4:** Comparison of Conventional PAWEC and Proposed SWWEC.

Category	Conventional PAWEC	Proposed SWWEC
Proximity to shore installation	✓	✓
Watertight structure	✓	×
Reduced manufacturing and installation costs	×	✓
Integration with other renewable energy sources	×	✓
Implementation of a battery swapping station	×	✓
Preservation of ocean corals	×	✓
Information of the ocean current	✓	✓
Mid sea local charging station	×	✓
Standalone systems	×	✓

## Laboratory validation and result analysis

The dynamic model of the hybrid SWWEC is emulated in a real-time simulation environment using DSpace. The DSpace model is shown in Figure. It can be interfaced with the real world through its analog and digital I/Os. The DSpace being a user-friendly program in which it can be interfaced with the real world through its analog and digital I/Os and the ability to programme like Simulink blocks is chosen instead of programming a processor for the required application.

### Hardware configuration

To execute the emulated SWWEC using a motor-generator setup, it’s important to have speed feedback from the motor. This feedback informs the torque command necessary for driving the motor. The motor is operated in torque-controlled mode, while the generator can be managed to generate power at the highest efficiency. A PMSG is used for this application.

**Fig. 11:**
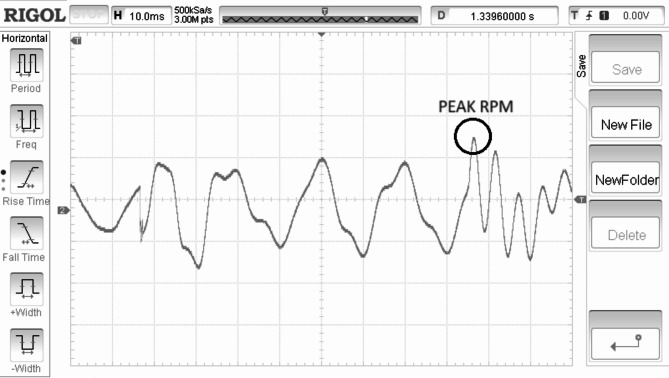
Generator Torque τ (1000/Div) (Nm).

**Fig. 12:**
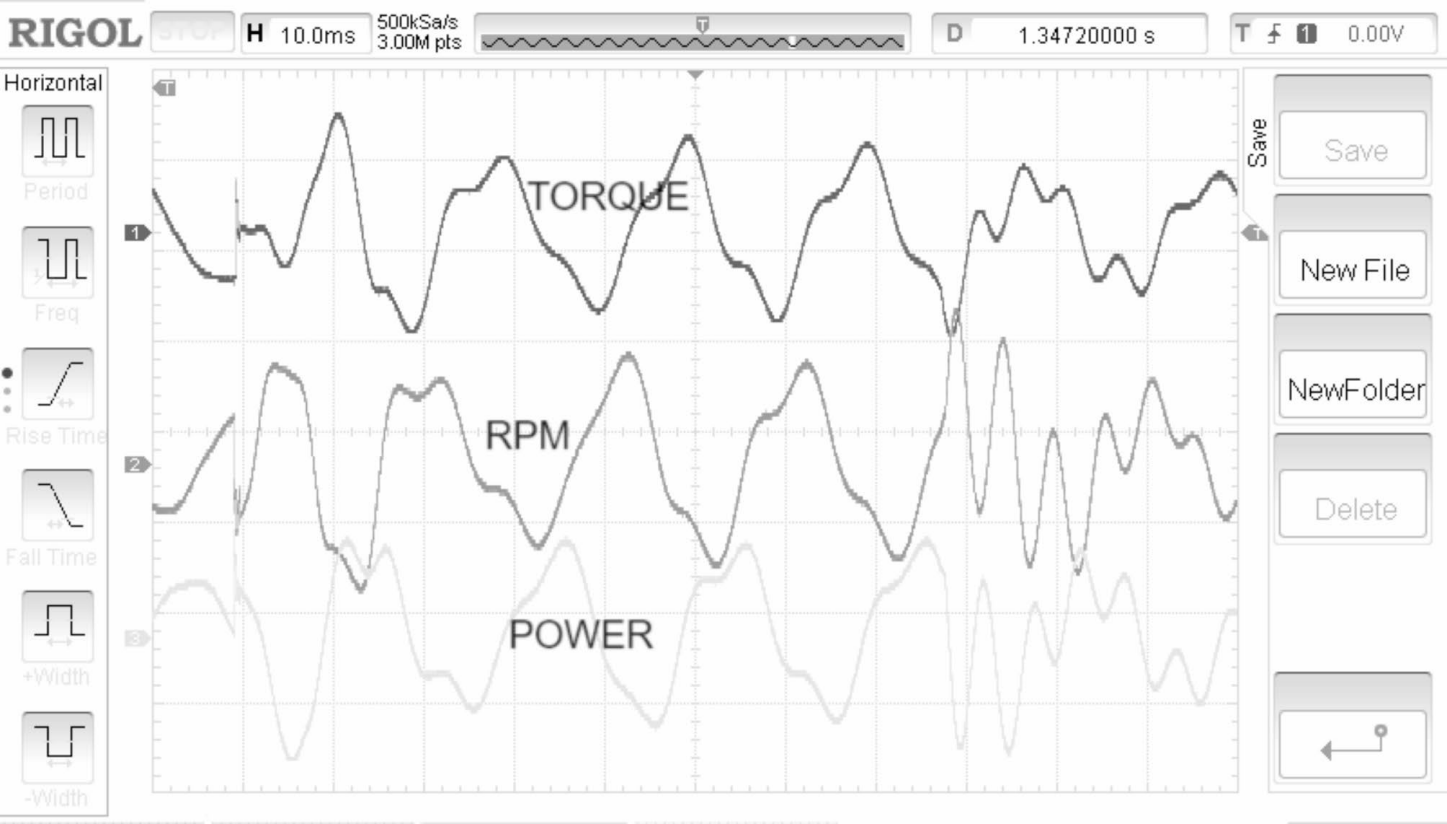
Rectified generator voltage (10/Div) (V).

**Fig. 13:**
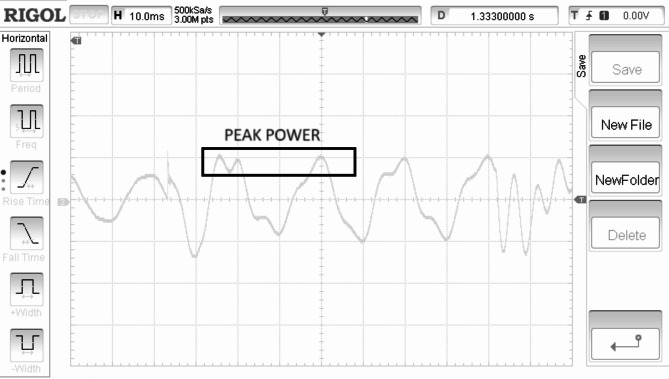
Rotor Speed ω (250/Div) (rad/sec).

**Fig. 14:**
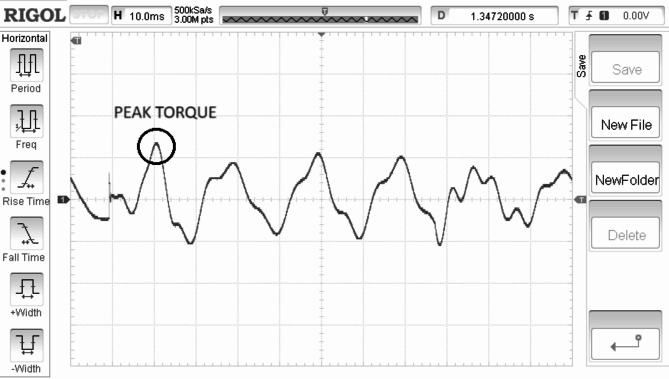
Generator Torque, Rotor Speed, Generator Power.

Electrical torque is regulated in direct relation to the speed of the rotor is shown in Fig. 11. Rectified generated voltage is shown in the Fig. 12. Rotor speed is for the SWWEC is presented in the Fig. 13 and the corresponding Generator Torque, Rotor Speed, Generator Power are shown in Fig. 14.

**Fig. 15:**
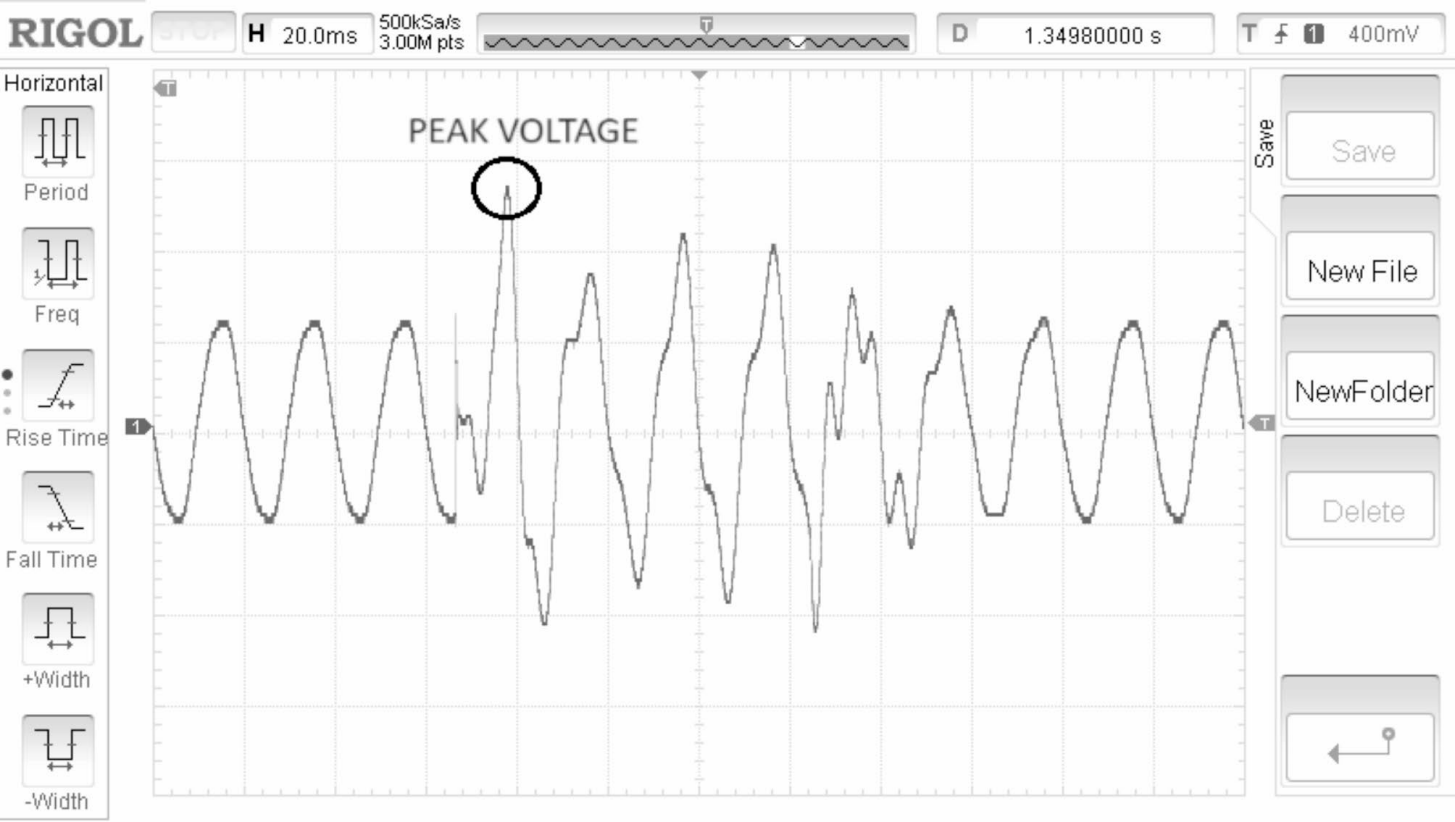
Generator Power (500/Div) (kW).

**Fig. 16:**
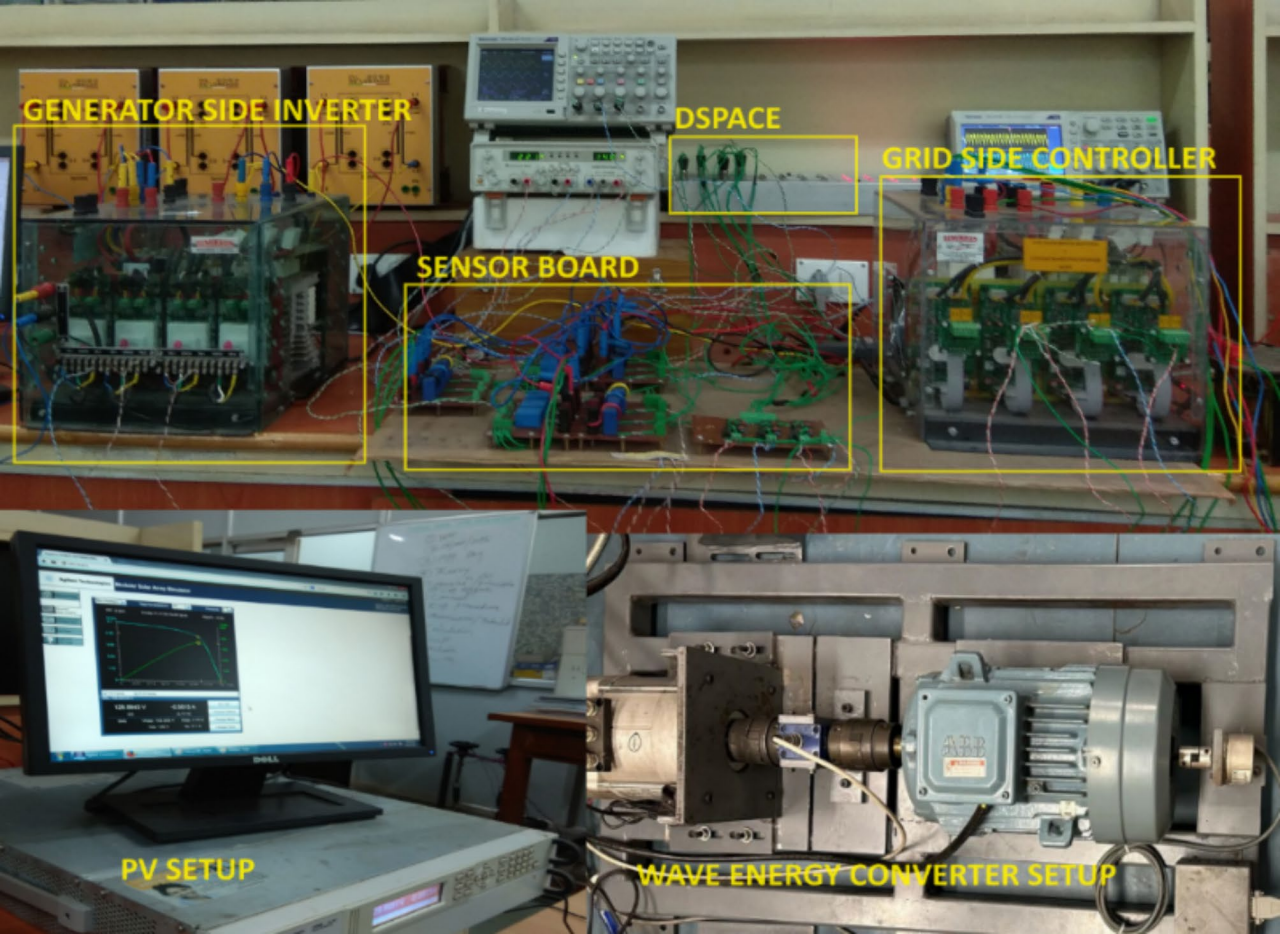
DSpace model, PV Setup & WEC Setup.

Figure 11 shows the hardware configuration. DSpace is responsible for supplying torque reference to the drive. The system is initially tested with a speed reference to demonstrate the oscillatory behaviour of the motor-generator setup. The emulating motor is speed controlled with sinusoidal speed reference and the generator output is shown in Figure. The highest frequency of 0.266 Hz of the sinusoidal component of the wave data is considered^27^. The SWWEC generated power in kW is from the laboratory prototype is shown in Fig. 15. Figure 16 shows the hardware configuration. DSpace is responsible for supplying torque reference to the drive. The system is initially tested with a speed reference to demonstrate the oscillatory behavior of the motor-generator setup. The emulating motor is speed controlled with sinusoidal speed reference and the generator output is shown in Figure.

The peak torque obtained during the experimentation was found to be 1200 Nm. The peak rectified generator voltage and generator power was found to be 38 V and 510 kW respectively at a maximum rotor speed of 290 rpm.

## Conclusion

The paper discusses dynamic emulation for a WEC along with a CAD model of the SWWEC, integrating solar, wind, and wave energy sources is proposed. The advantages of the SWWEC model over conventional PAWEC are highlighted. Additionally, real-time simulation results obtained using DSpace software are presented. The peak torque, rectified generator voltage, power and rotor speed obtained after the experimentation of the WEC are found to be 1200 Nm, 38 V, 510 kW and 290 rpm respectively. The emulation involves controlling an emulating motor using speed reference, with the results obtained of this control process are presented in the paper. To facilitate torque feedback-based emulation, the rotor speed needs to be fed back to the DSpace system. This feedback loop ensures that the emulation closely mimics the real-world behaviour of the WEC. In addition, the paper presents a comprehensive overview of WECs deployed worldwide along with their installed capacity. The objectives and conclusions drawn by various researchers across the world on different types of WECs are also compiled and presented for easy reference and comparison.

## Data Availability

The data used and/or analysed during the current study are available from the corresponding author upon reasonable request.

## Symbols

*A*: Area, $${m}^{2}$$
*a*: Radius of cylinder, m
*a*: Amplitude of wave, m
*B*: Arbitrary constant
*b*: Damping coefficient, kg/s
*c*: 976 Wm^-3^ s^-1^
*D*: Damping coefficient, kg/s
*D*: Diameter, m
*F*: Force, N
*f*: Frequency, Hz
*g*: Gravitational acceleration, $$m/{s}^{2}$$
*H*: Height of the wave, m
*h*: Depth, m
*i*: $$\sqrt{-1}$$
*k*_*e*_: Energy period, s
*m*: Mass, kg
*m*_*n*_: Spectral moment
*m*_*0*_: Spectral energy function
*P*: Power, W
*P*: Pressure, Pa
*Re*: Real part
*p*: Apparent power, VA
*S*: Restoring force coefficient, N/m
*S*_*f*_: Spectrum
*T*: Period, s
*W*: Watts
*W*: Annually produced energy
x: Acceleration, $$m/{s}^{2}$$

## Greek symbols

α: Angle, rad or degree
$$\alpha$$: Arbitrary constant
$\alpha$: Displacement in x direction, m
$$\alpha$$: Utility factor
$$\Delta$$: Damping ratio
$\delta$: Phase angle, rad or degree
$\epsilon$: Phase angle, rad or degree
$\theta$: Angle, rad or degree
$$\pi$$: 3.14159
$$\rho$$: Density, kg/$${\text{m}}^{3}$$
$\omega$: Angular velocity, rad/s
$$\gamma$$: Damping function
$$\tau$$: Torque, Nm

## Subscripts

abs: Absorbed
d: Damped
e: Energy
em: Electromagnetic inductive force
es: End stop
f: Friction
L: Loss
o: Resonant
p: Phase
p: Peak wave
r: Radiated
s: Significant
z: Zero-up crossing

## Abbreviations

AC: Alternating current
ANN: Artificial neural networks
ARO: Artificial rabbits optimization
AWS: Archimedes wave swing
CS: Cuckoo search
DC: Direct current
MPPT: Maximum power point tracking
OBWEC: Oscillating-body wave energy converter
OWCWEC: Oscillating water column wave energy converter
PAWEC: Point absorber wave energy converter
PMSG: Permanent-magnet synchronous generator
PV: Photovoltaic
PQ: Power quality
SBWEC: Sealed-buoy wave energy converter
SWWEC: Solar-wind-wave energy converter
UPQC: Unified power quality conditioners
VAWT: Vertical axis wind turbine
VQ: Voltage quality
WEC: Wave energy converter
WHO: Wild horse optimizer
